# Progress in Materials for Metallic Cultural Heritage Conservation: Mechanisms, Applications, and Future Perspectives

**DOI:** 10.3390/polym18091131

**Published:** 2026-05-04

**Authors:** Yutong Liu, Xiang Liu, Shanxiang Xu, Xinyou Liu

**Affiliations:** 1 College of Furnishing and Industrial Design, Nanjing Forestry University, Nanjing 210037, China; 2311404209@njfu.edu.cn (Y.L.); liuxiang@njfu.edu.cn (X.L.); xushanxiang@njfu.edu.cn (S.X.); 2 Co-Innovation Center of Efficient Processing and Utilization of Forest Resources, Nanjing Forestry University, Nanjing 210037, China

**Keywords:** metallic cultural heritage, corrosion mechanisms, novel functional materials, MOF composites, heritage conservation

## Abstract

Metallic cultural heritage artifacts are highly susceptible to multi-factor electrochemical degradation, driven by chloride ions, humidity, acidic deposition, and heterogeneous material interfaces. Traditional conservation materials, including organic and inorganic coatings and corrosion inhibitors, often exhibit limited interfacial compatibility, poor long-term stability, and insufficient multifunctionality. Recent advances in protective materials—including nano-enhanced coatings, self-healing systems, smart-responsive polymers, green biodegradable formulations, and metal–organic framework (MOF)-based composites—offer multifunctional, long-lasting, and minimally invasive solutions. These materials enhance corrosion inhibition, barrier performance, structural reinforcement, and environmental responsiveness, while enabling in situ sensing, reversible application, and ethical deployment. Laboratory evaluation, accelerated aging tests, and field verification demonstrate their efficacy in preserving artifact integrity and aesthetics. This review systematically discusses degradation mechanisms, limitations of traditional materials, and the mechanisms, applications, and future perspectives of novel functional coatings, providing a roadmap for scientifically optimized and ethically responsible conservation of metallic heritage.

## 1. Introduction

Metallic cultural heritage represents an irreplaceable physical carrier of human civilization, encompassing a wide range of objects, including bronze ritual vessels, iron artifacts, gold and silver ornaments, lead–tin alloy components, and metallic parts in modern technological relics, all of which carry historical, artistic, technological, and archaeological value [1]. During long-term burial, museum storage, exhibition, or exposure to outdoor environments, metallic artifacts are prone to irreversible degradation dominated by electrochemical corrosion, such as chloride-induced bronze disease, pitting, intergranular corrosion, and structural powdering [2,3]. Moreover, volatile pollutants emitted from display cases and protective materials, including formic acid and acetic acid, can accelerate the corrosion of lead, iron, copper, and their alloys, causing surface deterioration, information loss, and even complete structural failure [4,5].

Traditional conservation approaches primarily rely on mechanical cleaning, chemical rust removal, and natural protective materials (e.g., wax and shellac). While these methods are well-established and operable, they exhibit clear limitations: chemical rust removal may excessively erode the artifact substrate, and conventional protective materials suffer from poor aging resistance, insufficient breathability, and are prone to yellowing or cracking; additionally, harmful interfacial reactions may occur [2,6]. In highly saline, humid, and multi-pollutant environments, traditional materials often fail to satisfy modern conservation principles, which emphasize minimal intervention, reversibility, and long-term stability.

Recent studies indicate that polymer coatings, such as acrylics and polyurethanes, release acidic degradation products during photo-thermal aging, establishing a vicious cycle of “coating aging–accelerated metal corrosion–coating failure” [2,3]. Modern characterization techniques—including Fourier transform infrared spectroscopy (FTIR), Raman spectroscopy, GC-MS, scanning electron microscopy–energy-dispersive X-ray spectroscopy (SEM-EDS), X-ray diffraction (XRD), and colorimetric analysis—enable precise identification of artifact materials, corrosion products, and interfacial behaviors [7,8]. Concurrently, rapid advances in nanocomposites, organic–inorganic hybrid coatings, green corrosion inhibitors, and smart responsive materials provide targeted, environmentally friendly, breathable, reversible, and removable solutions for metal conservation [1,6,9].

Within this context, a systematic review of metallic artifact degradation mechanisms, limitations of traditional methods, and progress in novel protective materials is of significant theoretical and practical value. Continuous fiber-reinforced composites and 3D printing technologies offer new pathways for structural reinforcement and high-precision replication of artifacts [2,10]. Organic–inorganic ORMOSIL coatings, with their superhydrophilicity, underwater superoleophobicity, and anti-biofouling properties, provide long-lasting protection for multi-material artifacts [11,12]. Microenvironment-responsive nanoparticles can detect early corrosion signals and autonomously release inhibitors, laying the foundation for intelligent protection strategies [13]. The deepening of conservation ethics, authenticity principles, and minimal intervention approaches further drives the development of protective materials toward greener, reversible, and multifunctional directions [7,13].

**Figure 1:**
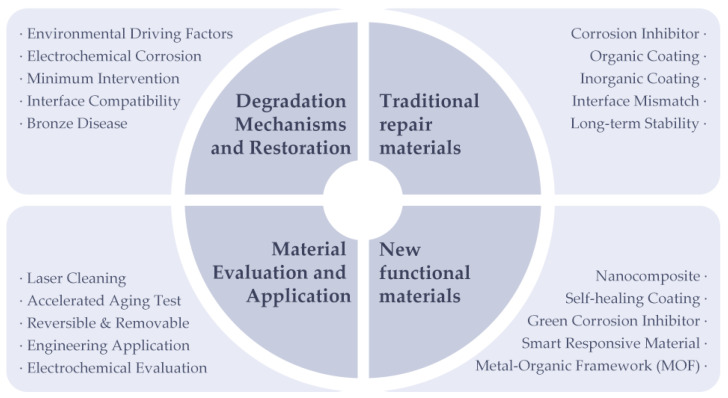
Research structure.

This review focuses on protective material systems for metallic cultural heritage, summarizing corrosion-driving mechanisms, the working principles and applications of both traditional and modern conservation materials, and analyzing key challenges in material selection, interfacial evaluation, and engineering applications. Furthermore, the review discusses future development trends, including green chemistry, smart responsive behavior, multifunctionality, and long-term reversibility, aiming to provide scientific guidance for the development and practical implementation of metallic artifact conservation materials. The overall structure of this review is illustrated in Figure 1.

## 2. Degradation Mechanisms of Metallic Cultural Heritage and Conservation Requirements

Metallic cultural heritage artifacts, whether buried, stored in museums, exposed outdoors, or incorporated into multi-material composite objects, commonly undergo irreversible degradation primarily driven by electrochemical corrosion, often coupled with multiple environmental stressors. The corrosion pathways, evolution of degradation products, and interfacial behaviors directly define the design logic and performance boundaries of protective materials. Building upon the introduction, this chapter systematically reviews typical degradation mechanisms, multidimensional environmental drivers, and outlines the core performance requirements for conservation materials based on modern preservation principles, providing a theoretical foundation for the subsequent discussion of novel protective material systems [14,15].

### 2.1. Degradation Mechanisms

**Figure 2:**
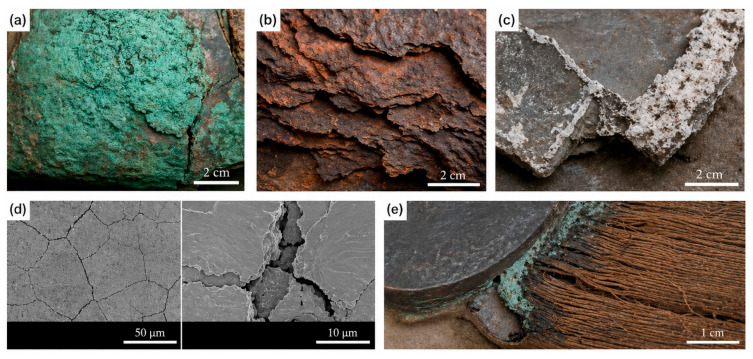
Representative corrosion morphologies of metallic cultural heritage materials. (**a**) Bronze disease on copper alloy artifacts showing green powdery Cu-based corrosion products; (**b**) Layered rust formation and powdering deterioration of iron artifacts; (**c**) White corrosion products formed on lead–tin alloys under organic acid exposure; (**d**) Intergranular corrosion and crack propagation in stainless steel components; (**e**) Interface corrosion in multi-material artifacts caused by galvanic interactions and volatile organic compounds. Note: Representative corrosion images can be adapted from Refs. [3,4,14,16,17,19,20,21], or alternatively redrawn as schematic diagrams to avoid copyright concerns.

The degradation of metallic cultural heritage is primarily governed by electrochemical corrosion processes, which vary significantly depending on alloy composition, burial history, environmental exposure, and interfacial heterogeneity. Different metallic substrates exhibit distinct corrosion pathways, degradation products, and failure morphologies, which directly influence the design requirements of conservation materials. To improve structural clarity and facilitate comparison, the representative degradation mechanisms of major metallic heritage materials are summarized in Table 1, while typical corrosion morphologies are presented in Figure 2.

As summarized in Table 1 and Figure 2, the degradation behavior of metallic cultural heritage materials is highly dependent on alloy composition, environmental pollutants, moisture conditions, and interfacial interactions. These differences directly determine the required corrosion resistance, compatibility, permeability, and reversibility of protective materials discussed in the following sections.

**Figure 3:**
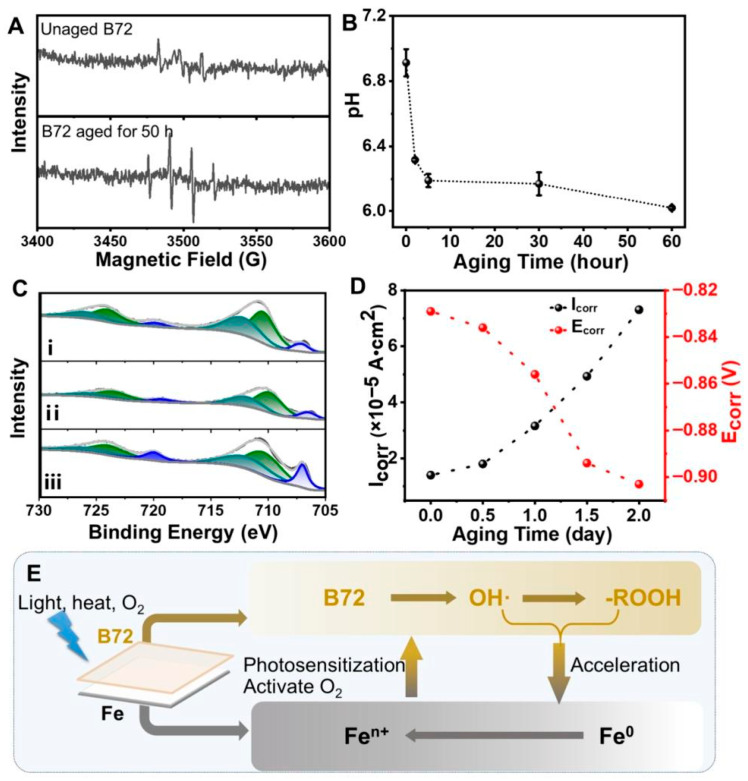
Influence of aged Paraloid B72 coating on the corrosion behavior of cast iron during aging exposure. (**A**) ESR spectra of unaged B72 and B72 aged for 50 h; (**B**) pH variation of leaching solutions from aged B72 films as a function of aging time (mean ± SD, n = 3); (**C**) XPS Fe 2p spectra of cast iron surfaces: (i) B72-coated and aged for 30 h (coating removed), (ii) bare cast iron aged for 30 h, and (iii) unaged cast iron, where blue, green, and gray lines represent Fe(0), Fe(II), and Fe(III), respectively; (**D**) evolution of corrosion current density (Icorr) and corrosion potential (Ecorr) of B72-coated cast iron during aging; (**E**) schematic illustration of the photothermal degradation of B72 and its interaction with the cast iron substrate [3].

As shown in Figure 3, the electrochemical behavior of cast iron coated with Paraloid B72 changes significantly after photothermal aging. The increase in corrosion current density (Icorr) after aging indicates progressive deterioration of the Paraloid B72 coating, which may be attributed to microcrack formation, increased porosity, and reduced barrier integrity during long-term environmental exposure. These structural defects facilitate the penetration of moisture, oxygen, and chloride ions to the metal/coating interface, thereby accelerating electrochemical corrosion reactions. In addition, the cathodic shift of corrosion potential (Ecorr) suggests increased electrochemical activity and localized corrosion susceptibility. Although freshly applied Paraloid B72 provides effective short-term protection, its long-term durability remains limited under harsh environmental conditions.

### 2.2. Environmental Drivers

The degradation of metallic cultural heritage artifacts results from the combined effects of heterogeneous substrate composition and external environmental stressors. Synergistic interactions among multiple factors can significantly accelerate corrosion rates and exacerbate structural damage. Corrosion mechanisms and rates vary considerably under different environmental conditions [22]. Among the primary drivers, indoor gaseous pollutants play a critical role: volatile organic compounds such as formic and acetic acids, released from museum display cases, wood, adhesives, and coatings, can markedly accelerate the corrosion of lead, copper, iron, and their alloys. In enclosed environments, elevated concentrations of these pollutants create localized acidic conditions that compromise passive films and induce pitting or crevice corrosion [4,19].

Temperature, humidity, and moisture similarly exert significant influence. When relative humidity exceeds 55%, a continuous water film forms on the metal surface, providing an electrolyte medium and causing the corrosion rate to increase exponentially. Fluctuating humidity promotes repeated dissolution and crystallization of salts, generating expansion stresses that destabilize corrosion product layers and induce structural powdering [23]. Soluble salts such as chloride ion (Cl^−^), sulfate ion (SO_4_^2−^), and nitrate ion (NO_3_^−^) also compromise metal passive films, triggering pitting and crevice corrosion. In marine or high-salinity archaeological sites, chloride ions are particularly critical in driving bronze disease and iron powdering [3,17].

Atmospheric and outdoor factors—including sulfur dioxide (SO_2_), nitrogen oxides (NO_x_), ozone (O_3_), hydrogen sulfide (H_2_S), and coastal sea-salt aerosols—form acidic deposits that accelerate the corrosion of steel and bronze structures, especially under prolonged exposure and weathering [19,22]. Microbial activity and biofilm formation are equally important; cyanobacteria, fungi, and other microorganisms can retain moisture and salts while metabolically producing acids, generating localized corrosive microenvironments that further accelerate degradation [24,25].

Under photothermal aging and mechanical loading conditions, ultraviolet and thermal radiation, along with cyclic mechanical stress, can accelerate the degradation of conventional organic coatings, causing cracking, powdering, and the release of acidic products. When coupled with fatigue loading, crack initiation and propagation rates are significantly increased [14,20]. Overall, metallic artifacts in complex environments exhibit multi-factor coupling in their degradation behavior, with mechanisms, corrosion products, and interfacial processes providing theoretical guidance for the design of conservation materials.

### 2.3. Performance Requirements for Conservation Materials

In accordance with modern heritage conservation principles, protective materials for metallic cultural heritage must not only mitigate corrosion but also preserve historical authenticity, reversibility, and long-term stability. Because metallic artifacts are exposed to highly heterogeneous environments, conservation materials are required to satisfy multiple functional criteria simultaneously. These requirements are summarized in Table 2.

The development of conservation materials has gradually shifted from traditional passive barrier coatings toward multifunctional systems that integrate corrosion inhibition, self-healing, environmental responsiveness, and condition monitoring. However, conventional materials often struggle to simultaneously satisfy all requirements listed in Table 3. These limitations have accelerated the development of micro/nanocontainer-based protective systems, which provide controlled release, intelligent response, and multifunctional protection strategies discussed in subsequent sections.

Overall, the degradation of metallic cultural heritage results from the combined effects of intrinsic material instability, external environmental stressors, and complex interfacial interactions. As summarized in Section 2.1, Section 2.2 and Section 2.3, different metallic substrates exhibit distinct corrosion pathways, while environmental heterogeneity further increases the difficulty of long-term preservation and imposes stringent requirements on conservation materials. Conventional protective systems often fail to simultaneously provide corrosion resistance, reversibility, adaptability, and long-term stability. These challenges highlight the necessity of developing advanced micro/nanocontainer-based conservation materials with intelligent release, self-healing, and multifunctional protection capabilities.

## 3. Traditional Metallic Conservation Materials

Traditional conservation materials have long served as the primary protective strategies for metallic cultural heritage, mainly through corrosion inhibition, physical barrier protection, and surface stabilization. These materials include corrosion inhibitors, organic coatings, inorganic coatings, and composite systems. Although they have demonstrated effectiveness in specific applications, their long-term performance remains limited by issues such as poor environmental adaptability, aging-induced failure, and insufficient multifunctionality. To provide a clearer comparison, the characteristics of traditional conservation materials are summarized in Table 3.

Based on the analysis of traditional conservation materials, the following conclusions can be drawn: although these systems provide basic corrosion inhibition and physical barrier protection, most remain passive and cannot effectively respond to dynamic environmental fluctuations or autonomously repair coating damage. Furthermore, challenges associated with reversibility, compatibility, aging resistance, and multifunctional integration remain unresolved. These limitations have accelerated the development of micro/nanocontainer-based conservation systems, which offer controlled release, intelligent responsiveness, and self-healing capabilities for advanced cultural heritage preservation.

## 4. Novel Functional Materials and Their Mechanisms of Action

To address the limitations of traditional metallic heritage conservation materials—including poor interfacial compatibility, susceptibility to aging and degradation, low protective efficiency, and irreversibility—novel functional materials have been developed with a focus on targeted corrosion inhibition, reversible protection, intelligent responsiveness, and environmental friendliness. Integrating insights from electrochemical corrosion, interface degradation, and multi-environment stressors, these materials have emerged into five major categories: nano-enhanced, self-healing, smart-responsive, green, and MOF-based composite systems [2,38]. They effectively address shortcomings of conventional materials in terms of permeability, barrier performance, interfacial adhesion, and multifunctional adaptability, providing a scientific basis for long-term protection of metallic artifacts.

### 4.1. Nanomaterials

**Figure 4:**
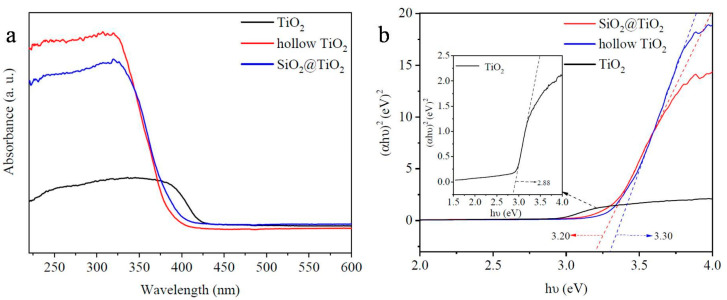
(**a**) Diffuse reflectance spectra and (**b**) band gap energy calculation of TiO_2_, SiO_2_@TiO_2_, and hollow TiO_2_ prepared at 850 °C [41], where the dashed lines in (**b**) represent the linear extrapolation used to determine the band gap energy.

Nanomaterials exploit size effects, high specific surface area, and strong interfacial adhesion to enhance corrosion inhibition, coating density, and structural reinforcement, making them essential for conserving fragile metallic heritage. Core–shell nanostructures such as SiO_2_@TiO_2_ combine gradient refractive index design with light-trapping effects (Figure 4), providing UV shielding, self-cleaning, and dense barrier properties that block O_2_, H_2_O, and Cl^−^ transmission. PDMS-OH and nano-SiO_2_ modified KH570-TEOS hybrids further improve coating hydrophobicity, mechanical strength, and aging resistance [39]. Similarly, nano-inhibitor particles such as Ce^3+^ and La^3+^ rare-earth oxides form passivating films via cathodic deposition, offering effective, non-toxic corrosion protection for bronzes and iron artifacts [28,29].

Two-dimensional materials, including graphene, black phosphorus, and h-BN, create ultrathin protective films that suppress electrochemical reactions through physical barriers and electronic modulation, with defect engineering enabling controlled ion transport for deep reinforcement and long-term corrosion inhibition. TiO_2_ nanoparticles exhibit anatase (A), brookite (B), and rutile (R) phases, providing high surface area and photoreactivity for self-cleaning and photocatalytic protection [40]. N-doping (N-TiA, N-TiU) extends visible-light response, enhancing photocatalytic activity under ambient light, while the crystal phase and doping optimize interfacial adhesion. These features make nanostructured TiO_2_ a non-invasive, durable strategy for protecting delicate stone or metallic cultural heritage objects.

As illustrated in Figure 4, various nanostructured materials, including core–shell nanoparticles and two-dimensional nanosheets, provide enhanced corrosion protection through barrier effects and interfacial interactions.

### 4.2. Self-Healing Coatings

Self-healing coatings actively repair microcracks, delay coating failure, and block interfacial corrosion, shifting protection from a “passive” to an “active” defense strategy. Microcapsule-based self-healing coatings encapsulate corrosion inhibitors—such as BTA derivatives, rare-earth salts, or plant polyphenols—within silica or polyurea microcapsules; upon coating damage, the inhibitors are released in situ to achieve localized passivation and repair. Intrinsic self-healing systems rely on dynamic covalent bonds (e.g., disulfide or acylhydrazone bonds), hydrogen bonding, or metal–ligand coordination, allowing molecular chain rearrangement and autonomous crack closure under humidity, temperature, or light stimuli. These systems exhibit reversible and repeatable healing capabilities [2,29]. The mechanisms involve crack-triggered inhibitor release, dynamic bond reorganization, and interfacial repassivation, forming a “damage–detection–repair–inhibition” loop that significantly extends the protective lifetime of the coating.

### 4.3. Smart-Responsive Materials

Smart-responsive materials sense environmental changes—such as pH, chloride ion concentration, humidity, or corrosion potential—and trigger protective actions, enabling precise, on-demand, and controllable corrosion mitigation. pH-responsive materials swell or degrade in acidic microenvironments, releasing inhibitors directionally to provide immediate corrosion suppression. Ion-responsive materials detect corrosive species like Cl^−^ or SO_4_^2−^, activating colorimetric changes or inhibitor release. Homogeneous, non-immobilized electrochemiluminescent sensors leverage electrostatic interactions and nuclease-based signal amplification for ultra-sensitive corrosion monitoring, while functionalized Cu-MOF fluorescent sensors allow rapid quantitative detection of corrosion agents [21,42]. The overall mechanisms include environmental signal recognition, molecular conformational change, controlled inhibitor release, and interfacial potential regulation, integrating early warning, corrosion inhibition, and repair into a single responsive system.

### 4.4. Green and Sustainable Materials

Green materials prioritize low toxicity, degradability, reversibility, removability, and absence of secondary pollution, providing alternatives to toxic inhibitors and hard-to-remove resins. Bio-based inhibitors—including plant polyphenols (e.g., tannins, tea polyphenols), amino acids, polysaccharides, and plant extracts—achieve corrosion inhibition through multi-functional physical adsorption or chemical coordination with metal surfaces [4,31]. Waterborne systems such as acrylics, polyurethanes, gelatin microspheres, and chitosan composites offer low toxicity, high permeability, and mild removability, making them compatible with museum display environments. Ormosil organic–inorganic hybrid coatings combine superhydrophilicity, underwater superoleophobicity, and anti-biofouling properties, while bio-based materials like chitosan and gelatin microspheres provide inherent reversibility and biocompatibility [24,43]. Their mechanisms include multi-site adsorption and film formation, biocompatible interfacial interactions, and degradable/reversible removal, effectively preventing damage from acidic degradation products or toxic substances.

### 4.5. MOF-Based and Composite Materials

Metal–organic frameworks (MOFs) have become a new generation of multifunctional protective carriers due to their ultra-high surface area, tunable porosity, functional modifiability, and controllable loading. They allow integrated corrosion inhibition, adsorption, controlled release, and sensing [21,44]. MOF-based inhibitor carriers, such as UiO-66, ZIF-8, and MIL series, can load corrosion inhibitors, plant polyphenols, and rare-earth ions for sustained release to suppress electrochemical corrosion. PtNPs@Co(II)MOF combines high catalytic activity with intrinsic redox properties, strengthening both electrochemical response and inhibition efficiency [43].

**Figure 5:**
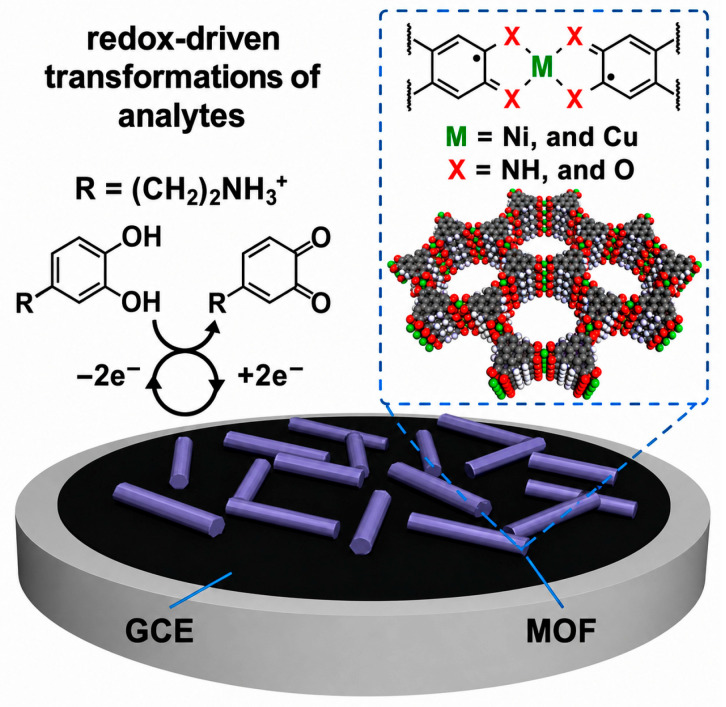
Schematic illustration of a metal–organic framework (MOF)-modified glassy carbon electrode (GCE), depicting redox-driven transformations of analytes at the electrode interface and the coordination environment of the MOF. Metal centers (M = Ni, Cu) and coordinating heteroatoms (X = N, O) are indicated by green and red spheres, respectively.

**Figure 6:**
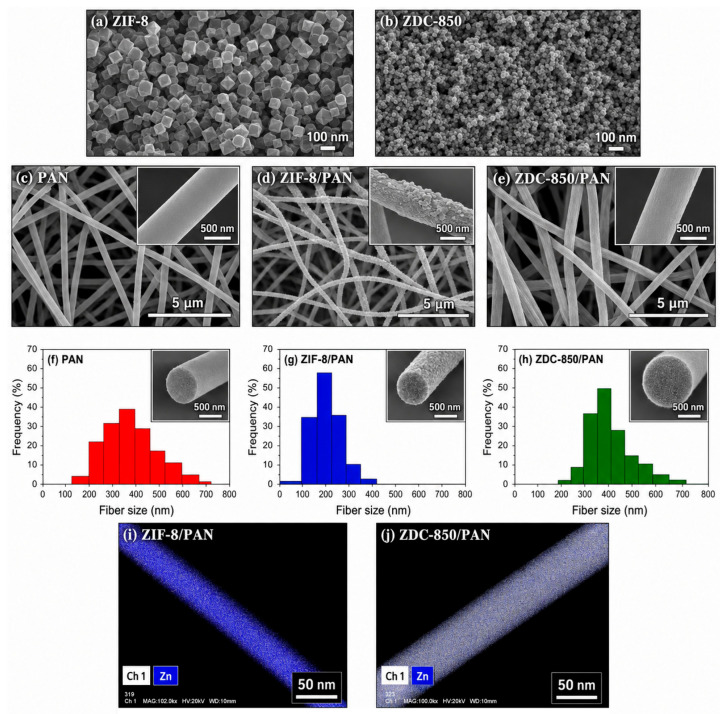
FESEM images, fiber diameter distributions, and HRTEM–EDX elemental mapping of the samples: (**a**) ZIF-8 nanoparticles; (**b**) ZDC-850 nanoparticles; (**c**) PAN nanofibers; (**d**) ZIF-8/PAN composite nanofibers; (**e**) ZDC-850/PAN composite nanofibers; (**f**–**h**) corresponding fiber diameter distributions of PAN, ZIF-8/PAN, and ZDC-850/PAN, respectively; (**i**) HRTEM–EDX mapping of ZIF-8/PAN nanofibers showing Zn distribution; (**j**) HRTEM–EDX mapping of ZDC-850/PAN nanofibers, indicating the elemental distribution within the carbonized composite [45].

**Figure 7:**
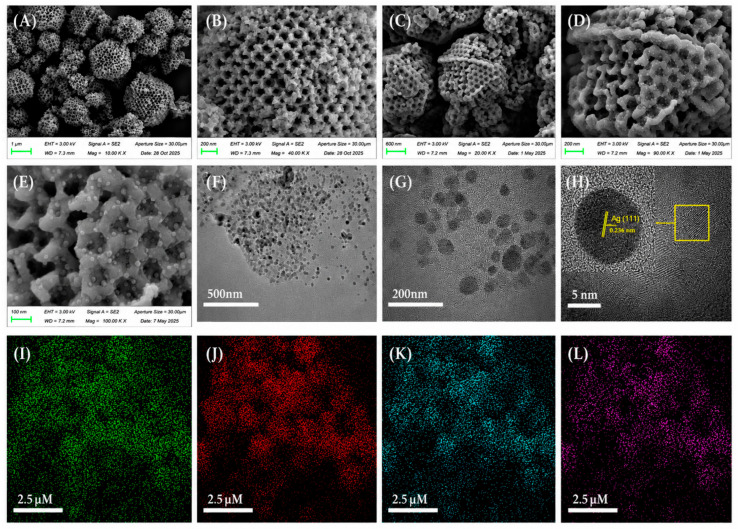
Morphological and structural characterization of ZIF-8-based composites. (**A**,**B**) SEM images of SOM-ZIF-8; (**C**–**E**) SEM images of Ag-SOM-ZIF-8; (**F**) high-angle annular dark-field scanning transmission electron microscopy (HAADF-STEM) image of Ag-SOM-ZIF-8; (**G**,**H**) high-resolution transmission electron microscopy (HRTEM) images of Ag-SOM-ZIF-8, where the yellow box in (**H**) delineates the selected region of interest (ROI) for localized lattice/structural analysis; (**I**–**L**) energy-dispersive X-ray spectroscopy (EDS) elemental mapping of Ag-SOM-ZIF-8, illustrating the spatial distribution of constituent elements [46].

MOFs also regulate microenvironments by capturing volatile acids (formic and acetic acid) and chloride ions, reducing bronze disease and corrosion risk. MOF composite coatings combined with organic–inorganic hybrids, carbon nanotubes, or graphene enhance coating density, interface adhesion, and aging resistance. Flexible Ag/ZIF-8/PAN SERS substrates allow in situ detection of surface pollutants and corrosion products [21,44] (Figure 5, Figure 6 and Figure 7). Mechanisms include pore-confined loading, stimulus-responsive release, harmful ion/gas adsorption, interface coordination, and synergistic barrier effects, providing a multifunctional, long-lasting, and minimally invasive protective solution for metallic heritage artifacts.

Figure 5, Figure 6 and Figure 7 systematically characterize the microstructure, porosity, and elemental composition of MOF-based composite materials for metallic heritage conservation. Figure 5 illustrates the fabrication and electrochemical performance of a glassy carbon electrode (GCE) modified with MOF materials, where cyclic voltammetry tests reveal distinct redox peaks and favorable quantitative response, enabling sensitive in situ detection of corrosion-related species. Figure 6 shows the N_2_ adsorption–desorption isotherms and pore-size distribution of PAN nanofibers, ZIF-8 nanoparticles, and ZIF-8/PAN composites; Brunauer–Emmett–Teller (BET) analysis confirms that the composite possesses large specific surface area and rich porosity, which support high-capacity loading and sustained release of corrosion inhibitors. Figure 7 presents the XPS results of Ag/ZIF-8/PAN nanofibers, in which characteristic signals of Ag, Zn, C, N, and O are detected, demonstrating uniform elemental distribution and successful preparation of the composite. These characterizations verify that MOF-based composites exhibit favorable porosity, structural uniformity, and electrochemical activity, making them promising candidates for multifunctional protection and in situ monitoring of metallic cultural heritage.

Overall, Section 4 demonstrates that micro/nanocontainer systems provide substantial advantages over traditional conservation materials by enabling controlled release, self-healing behavior, and environmental responsiveness. Different carrier systems—including microcapsules, mesoporous materials, layered nanostructures, and MOF-based composites—offer distinct functional benefits depending on the preservation scenario. However, challenges related to loading efficiency, long-term stability, material compatibility, and scalable fabrication remain important considerations for practical heritage applications.

## 5. Material Evaluation Methods and Practical Applications

To ensure the scientific assessment, long-term aging reliability, engineering applicability, and ethical compliance of metallic heritage conservation materials, a comprehensive evaluation framework has been established, encompassing performance testing—accelerated aging—on-site validation—reversibility and safety assessment [2,45,47]. This framework integrates recent advances in nano-SiO_2_/TiO_2_ hybrid coatings, ZnO/natural polymer composites, fluorinated polyacrylate coatings, Nd:YAG pulsed laser cleaning, and polymer aging–interface studies, providing standardized guidance for the selection and engineering application of nanocomposite, self-healing, smart-responsive, and MOF-based composite materials.

### 5.1. Material Performance Evaluation Methods

Material performance evaluation integrates electrochemical, microstructural, spectroscopic, macroscopic, and mechanical assessments to quantify corrosion inhibition, barrier effectiveness, and structural reinforcement. Electrochemical evaluation employs polarization curves, electrochemical impedance spectroscopy (EIS), and zero-resistance ammetry (ZRA) to measure corrosion current density (I_corr), corrosion potential (E_corr), and interfacial impedance, providing quantitative insight into inhibitor efficiency and coating barrier performance [31,33]. Studies show that nano-SiO_2_/fluorinated polyacrylate composites and ZnO-hybrid coatings can reduce corrosion rates to 0.026–0.067 mm/year in 3.5% NaCl solution, increasing interfacial impedance by 1–2 orders of magnitude, comparable to commercial Incralac coatings [45,48]. Microstructural and interfacial characterization via SEM–EDS, AFM, FTIR, XRD, and XPS reveals coating morphology, elemental distribution, functional groups, and corrosion products; ZnO nanoparticles in GPTMS–TEOS silica-based coatings are uniformly dispersed with surface roughness Ra < 1 nm, while nano-SiO_2_-modified coatings form dense maze-like networks that extend the diffusion path of corrosive media [46]. Spectroscopic and optical non-destructive techniques, including visible–shortwave infrared hyperspectral imaging, fluorescence spectroscopy, SERS, and laser-induced breakdown spectroscopy (LIBS), enable rapid detection of surface corrosion, contaminants, and protective materials, with Nd:YAG pulsed laser cleaning maintaining a color difference ΔE < 5, within acceptable thresholds for heritage objects [49,50]. Macroscopic physical performance tests evaluate adhesion, hardness, water contact angle, breathability, and anti-fouling capability; nano-SiO_2_/fluorinated polyacrylate coatings achieve water contact angles of 85–90° and meet adhesion Class 1 standards, providing balanced hydrophobicity and permeability suitable for artifact reinforcement [45]. Mechanical and fatigue assessments for large metal components and 3D-printed replicas, including tensile, bending, and fatigue tests, show that Cr_23_C_6_ precipitation in Super304H welded joints under high-temperature, low-strain conditions leads to localized hardening and fatigue failure transitioning from base material to weld, providing critical safety evaluation criteria for large-scale metal heritage structures [47,48].

### 5.2. Accelerated Aging Experiments

To assess the long-term reliability of metallic heritage conservation materials, accelerated aging experiments simulate environmental stressors such as UV irradiation, humidity cycling, temperature fluctuation, saline exposure, and acidic deposition. Electrochemical techniques—including polarization curves, electrochemical impedance spectroscopy (EIS), and zero-resistance ammetry (ZRA)—quantify corrosion current density (I_corr), corrosion potential (E_corr), and interfacial resistance, providing insights into inhibitor efficacy and coating barrier performance [40,51]. Hybrid nanocomposite coatings, such as nano-SiO_2_/fluorinated polyacrylate and ZnO-based systems, demonstrate corrosion rates reduced to 0.026–0.067 mm/year in 3.5% NaCl solutions and interfacial impedance increased by one to two orders of magnitude, comparable to commercial Incralac coatings. Structural and surface analyses using SEM–EDS, AFM, FTIR, XRD, and XPS reveal uniform nanoparticle dispersion and dense maze-like morphologies that extend corrosion-path tortuosity [29,51]. Spectroscopic and optical non-destructive methods, including hyperspectral imaging, SERS, fluorescence spectroscopy, and LIBS, monitor corrosion products, surface contaminants, and coating integrity. For instance, post-treatment Nd:YAG pulsed laser cleaning maintains ΔE < 5, ensuring no significant visual alteration [49,50]. Macroscopic tests such as adhesion, hardness, water contact angle, permeability, and fouling resistance further validate protective performance, with hybrid nanocomposites achieving contact angles of 85–90° and adhesion levels sufficient for artifact reinforcement [45]. Mechanical and fatigue testing on large metal artifacts or 3D-printed replicas confirms structural stability under service-like stress conditions [47,52].

### 5.3. Practical Application Case Studies

**Figure 8:**
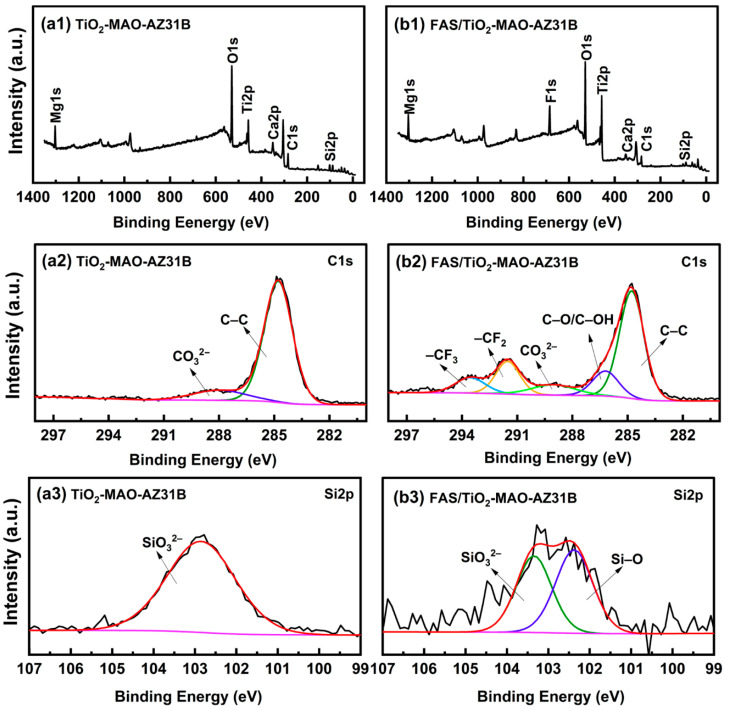
XPS characterization of TiO_2_-MAO and FAS-TiO_2_-MAO coatings: survey spectra (**a1**,**b1**), high-resolution C 1s spectra (**a2**,**b2**), and high-resolution Si 2p spectra (**a3**,**b3**) [49]. The black lines represent the experimental spectra, the red lines denote the fitted curves, and the colored peaks correspond to deconvoluted components.

Accelerated aging experiments and real-world deployment are critical for validating protective coatings intended for metallic cultural heritage. Accelerated aging tests simulate prolonged environmental exposure using UV irradiation, humidity cycles, and thermal fluctuations to assess coating durability and performance consistency. For instance, PFDTS-TiO_2_/SiO_2_ nano-coatings applied to sandstone substrates demonstrated that after 210 h of UV exposure, both water contact angles (WCAs) and oil contact angles (OCAs) decreased by less than 20%, confirming the long-term retention of amphiphobic properties under simulated environmental stress [53]. Gas permeability measurements verified that coatings do not hinder substrate “breathing”, preventing internal moisture damage, which is essential for preserving the physical integrity of artifacts (Figure 8(a1,b1)). SEM imaging revealed that the micro- and nanoscale rough structures of these coatings remain intact post-aging, ensuring continued self-cleaning and protective functionality (Figure 8(a2,b2)).

Practical field verification complements laboratory evaluation by deploying coatings on bronze, iron, and steel artifacts under museum and outdoor conditions. MOF-based composite coatings, hybrid fluorinated polyacrylate–SiO_2_ systems, and N-doped TiO_2_ films effectively suppress chloride-induced corrosion while maintaining aesthetic and structural integrity. In situ monitoring via electrochemical probes and SERS enables real-time performance assessment and environmental response tracking, providing predictive protection against micro-corrosion. Reversibility and conservation ethics are central to modern applications: coatings must be removable or degradable without leaving residues, avoiding chemical or mechanical interference with the original material. This integrated evaluation framework—from performance testing and accelerated aging to field verification and ethical assessment—ensures that novel functional coatings meet both scientific and heritage conservation requirements [21,29,53].

As presented in Figure 8, gas permeability tests were carried out on uncoated sandstone (Blank 1, Blank 2) and nano-coating treated sandstone (Sample 1, Sample 2). The mass–time curves and calculated permeability values show that the PFDTS-TiO_2_/SiO_2_ nano-coating maintains sufficient gas permeability while providing effective protection, which meets the “breathable” requirement for cultural relics conservation.

SEM images (Figure 8(a3)) reveal that the nano-coating forms a uniform and dense surface layer without obvious cracks. Energy-dispersive X-ray spectroscopy (EDX) analysis (Figure 8(b3)) confirms the presence of Ti, Si, and O derived from the nano-coating, verifying successful deposition of the protective layer. After UV aging, the coating retains favorable wettability and structural integrity, demonstrating good weathering resistance for long-term conservation use.

### 5.4. Reversibility and Ethical Considerations

Reversibility and minimal intervention remain core principles in conservation ethics. Protective coatings and reinforcements must be removable without damaging the artifact substrate, leaving no residues or altering surface morphology. Water-based, biodegradable, or polymeric coatings that allow gentle removal under controlled conditions meet these criteria [6,13]. Ethical deployment also demands the evaluation of toxicity, environmental impact, and long-term sustainability. The combination of laboratory testing, accelerated aging, and field validation ensures that applied materials satisfy both technical and ethical requirements, preserving cultural heritage in a scientifically responsible and minimally invasive manner.

**Figure 9:**
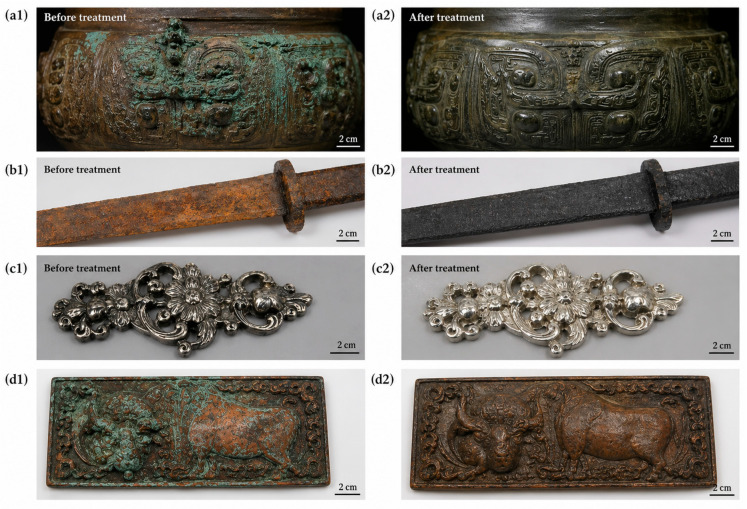
Representative practical applications of advanced conservation materials in metallic cultural heritage preservation. (**a1**,**a2**) Bronze artifact before and after corrosion stabilization treatment; (**b1**,**b2**) Iron artifact before and after protective coating treatment; (**c1**,**c2**) Silver artifact before and after anti-tarnish treatment; (**d1**,**d2**) Copper artifact before and after nano-coating protection treatment.

As shown in Figure 9, advanced conservation strategies enable effective stabilization and restoration of metallic artifacts, including bronze, iron, silver, and copper objects, through corrosion inhibition, protective coatings, and surface cleaning treatments. In summary, despite the significant progress of intelligent conservation materials, several practical barriers continue to limit their large-scale implementation in cultural heritage preservation. Challenges associated with long-term durability, environmental adaptability, ethical reversibility, and real-world validation must be systematically addressed before widespread adoption. Future research should emphasize interdisciplinary collaboration among materials scientists, conservators, museum professionals, and policy makers to ensure both technological innovation and heritage authenticity.

## 6. Challenges and Future Directions

Despite the significant progress in protective materials for metallic cultural heritage, several critical scientific and engineering challenges remain. Interfacial compatibility is still a major concern: multi-material artifacts often exhibit heterogeneous interfaces, and even advanced nanocomposite or MOF-based coatings may experience delamination or local stress accumulation, leading to suboptimal adhesion and uneven protection. Long-term stability under coupled environmental stressors—such as UV radiation, humidity, acidic deposition, and saline exposure—remains limited for many coatings, including polymeric, hybrid, and inorganic systems. Aging-induced degradation can release acidic byproducts, accelerating underlying metal corrosion, thereby creating a feedback loop that undermines protective efficacy [3,51].

Smart-responsive and self-healing materials face challenges in precision and controllability. The kinetics of inhibitor release or crack healing may not synchronize with environmental triggers, limiting early corrosion suppression. Similarly, MOF-based systems require careful tuning of pore size, loading, and release kinetics to achieve predictable performance, especially under fluctuating environmental conditions [2,54]. Green chemistry, reversibility, and multifunctionality also present inherent trade-offs: biocompatible and low-toxicity materials may compromise mechanical strength, barrier performance, or long-term durability. Current evaluation methods, although comprehensive in the lab, often fail to fully replicate in situ conditions, highlighting the need for predictive, multi-factorial testing and monitoring frameworks [55,56].

Future development should focus on five key directions: (1) atomic-scale interfacial design to improve adhesion and prevent local delamination; (2) multifunctional integration combining corrosion inhibition, structural reinforcement, self-cleaning, and environmental responsiveness; (3) intelligent, self-adaptive systems capable of early warning, controlled inhibitor release, and autonomous repair; (4) green and reversible materials with minimal environmental impact and compatibility with conservation ethics; and (5) digital and predictive evaluation frameworks, integrating electrochemical sensing, spectroscopy, and machine learning for real-time monitoring and performance prediction. The convergence of these strategies aims to transform metallic heritage conservation from empirical treatment to scientifically guided, minimally invasive, long-term protection.

## 7. Conclusions

This review summarizes the mechanisms driving metallic artifact degradation, the limitations of traditional protective materials, and the development of novel functional coatings and composites. Metallic artifacts degrade through multi-factor electrochemical processes influenced by chloride ions, humidity, acidic deposition, and heterogeneous material interfaces, underscoring the need for materials with precise interfacial control and effective barrier properties. Traditional conservation materials—including organic and inorganic coatings and corrosion inhibitors—exhibit limited interfacial compatibility, poor long-term stability, and insufficient multifunctionality, highlighting the need for next-generation solutions. Novel materials, such as nano-enhanced coatings, self-healing systems, smart-responsive polymers, green and biodegradable formulations, and MOF-based composites, provide multifunctional, long-lasting, and minimally invasive protection. In particular, MOFs enable integrated corrosion inhibition, controlled release, adsorption of harmful ions, and in situ sensing, enhancing both preventive and responsive strategies. Accelerated aging studies, laboratory testing, and field deployment demonstrate that these advanced materials effectively maintain protective function, aesthetic integrity, and environmental compatibility, while reversibility and ethical considerations remain central to their practical adoption. Future conservation strategies should focus on interfacial engineering, multifunctional integration, intelligent adaptation, green chemistry, and predictive evaluation frameworks to ensure durable, ethically responsible, and scientifically optimized protection of metallic cultural heritage. Collectively, these insights provide a roadmap for developing, assessing, and applying advanced materials that bridge fundamental research with practical implementation in heritage conservation.

## Figures and Tables

**Table 1:** Typical degradation pathways of metallic cultural heritage materials.

Material	Degradation Mechanisms	Examples of Damage (with Photos)	Representative References
Bronze and copper alloys	Chloride-induced corrosion (bronze disease), CuCl hydrolysis, oxidation, patina destabilization	Green powdery corrosion products, pitting corrosion, surface cracking (Figure 2a)	[16,17]
Iron-based artifacts	Electrochemical oxidation (Fe → Fe^2+^ → Fe^3+^), chloride-assisted corrosion, differential aeration corrosion	Layered rust formation, delamination, structural powdering (Figure 2b)	[3,18]
Lead–tin alloys	Organic acid corrosion, oxidation, recrystallization embrittlement	White corrosion crusts, surface whitening, powdering (Figure 2c)	[4,19]
Stainless steel and modern metallic components	Pitting corrosion, intergranular corrosion, stress corrosion cracking, fatigue corrosion	Localized pits, grain boundary cracking (Figure 2d)	[14,20]
Multi-material composite artifacts	Galvanic corrosion, volatile organic compounds (VOCs)-induced corrosion, interface degradation, microbial corrosion	Metal/wood/textile interface corrosion, localized deposits (Figure 2e)	[3,17]

**Table 2:** Performance requirements for conservation materials used in metallic cultural heritage preservation.

Performance Requirement	Functional Purpose	Typical Challenges	Representative Strategies/Materials	Representative References
Corrosion resistance	Prevent penetration of moisture, oxygen, chlorides, and pollutants	Coating degradation in aggressive environments	Epoxy coatings, fluorinated polymers, sol-gel barriers	[3,16,17]
Reversibility	Ensure future removal without damaging original substrates	Excessive adhesion may hinder retreatment	Paraloid B72, reversible acrylic coatings	[13,26]
Compatibility	Avoid adverse chemical interactions with original materials	pH mismatch, galvanic incompatibility	Neutral polymers, corrosion inhibitors	[4,14,19]
Mechanical durability	Resist cracking, abrasion, and deformation	Brittle coatings under thermal/mechanical stress	Hybrid nanocomposites, flexible polymers	[20,27]
Optical transparency	Preserve visual appearance and surface details	Yellowing, opacity, gloss alteration	Acrylic coatings, silica coatings	[13,22]
Environmental responsiveness	Adapt to humidity, pH, chloride fluctuations	Conventional coatings are passive	Stimuli-responsive microcapsules, smart coatings	[21,28,29]
Long-term stability	Maintain protection during aging	UV degradation, oxidation, hydrolysis	UV-resistant polymers, hybrid materials	[3,17,30]
Sustainability and safety	Reduce toxicity and environmental hazards	Toxic inhibitors, VOC emissions	Bio-based polymers, green inhibitors	[24,31,32]
Monitoring capability	Enable early corrosion detection	Lack of diagnostic functionality	Fluorescent sensors, self-reporting coatings	[21,33,34]
Storage and exhibition adaptability	Protect artifacts during transport/storage/display	Variable museum environments	Smart packaging materials, adaptive coatings	[24,25,35]

**Table 3:** Comparison of traditional conservation materials for metallic heritage preservation.

Type of Material	Main Representatives	Mechanism of Action	Advantages	Disadvantages	Representative References
Corrosion inhibitors	Benzotriazole (BTA), tannic acid, rare-earth inhibitors, amino acid inhibitors	Adsorption on metal surfaces, passivation layer formation, suppression of electrochemical reactions	High inhibition efficiency, easy application, relatively low cost	Toxicity concerns, discoloration risk, limited long-term stability	[4,16,36]
Organic coatings	Paraloid B72, acrylic resins, epoxy resins, polyurethane coatings, wax coatings	Formation of physical barriers against oxygen, moisture, and pollutants	Good transparency, strong adhesion, easy processing	Aging, yellowing, cracking, poor reversibility	[3,13,17]
Inorganic coatings	Sol-gel coatings, silica coatings, phosphate coatings, layered double hydroxides	Dense barrier formation and chemical stabilization	High thermal stability, excellent corrosion resistance	Brittleness, poor flexibility, difficult repair	[20,37,38]
Hybrid composite coatings	Organic–inorganic hybrids, nanoparticle-reinforced coatings	Combined barrier protection and mechanical reinforcement	Improved durability, multifunctionality	Complex fabrication, relatively high cost	[25,27]
Temporary storage materials	Desiccants, oxygen scavengers, corrosion adsorbents, protective packaging materials	Environmental regulation during storage and transportation	Easy implementation in museums	Limited direct corrosion inhibition	[21,24,35]
Monitoring materials	pH indicators, fluorescence probes, corrosion sensors	Early detection of corrosion and environmental fluctuations	Real-time monitoring potential	Limited practical deployment	[21,31,34]

## Data Availability

No new data were created or analyzed in this study. Data sharing is not applicable to this article.
